# Unlocking Niclosamide Solid Forms via Mechanochemistry: Discovery of a 2-Pyrrolidone Solvate

**DOI:** 10.3390/pharmaceutics18080906

**Published:** 2026-07-23

**Authors:** Ilenia D’Abbrunzo, Veronica Rizzetto, Lara Gigli, Nicola Demitri, Francesca Argentieri, Mauro Stener, Nadia Passerini, Beatrice Perissutti

**Affiliations:** 1 Department of Chemical and Pharmaceutical Sciences, University of Trieste, P.le Europa 1, 34127 Trieste, Italyfrancesca.argentieri@phd.units.it (F.A.); stener@units.it (M.S.); 2 Area Science Park Elettra-Sincrotrone Trieste, S.S. 14 Km 163.5 Basovizza, 34149 Trieste, Italy; lara.gigli@elettra.eu (L.G.); nicola.demitri@elettra.eu (N.D.); 3 Department of Pharmacy and Biotechnology, University of Bologna, V. S. Donato 19/2, 40127 Bologna, Italy; nadia.passerini@unibo.it

**Keywords:** niclosamide, 2-pyrrolidone, mechanochemistry, niclosamide monosolvate, niclosamide monohydrate

## Abstract

**Background/Objectives**: Niclosamide, an anthelmintic drug included in the World Health Organization Model List of Essential Medicines, has recently attracted attention for drug repurposing applications. Its strong tendency to form solvates and hydrates also makes it an attractive candidate for solid-state investigations. This study aimed to evaluate mechanochemical screening as a tool for solvate discovery and to characterize the resulting solid forms. **Methods**: Mechanochemical screening was performed using twelve selected solvents commonly employed in pharmaceutical research and solid-state investigations and compared with slurry experiments. The obtained phases were characterized by PXRD, DSC, TGA, FTIR, HSM, and SEM. The crystal structure of the new phase was solved from PXRD data and validated by DFT calculations. Stability and kinetic solubility studies were conducted to assess the properties of the identified solid forms. **Results**: The screening demonstrated the effectiveness of mechanochemistry as a rapid and resource-efficient approach for identifying solvate-forming solvents. Comprehensive characterization clarified the influence of the selected solvents on the solid-state landscape of niclosamide and their relationship with previously reported phases. A new 2-pyrrolidone solvate was discovered and structurally characterized as a triclinic *P*-1 phase with a 1:1 niclosamide:2-pyrrolidone stoichiometry. The new solvate exhibited distinctive thermal behavior, remained stable for at least 18 months, and showed mechanical robustness under compression. Furthermore, it displayed delayed conversion to niclosamide H_A_ monohydrate in water and enhanced resistance to solid-state transformation under humid conditions. **Conclusions**: Mechanochemical screening represents an efficient strategy for exploring the solvate landscape of niclosamide. The newly discovered 2-pyrrolidone solvate combines structural stability and favorable solid-state performance, highlighting its potential relevance for future pharmaceutical development.

## 1. Introduction

**Figure 1:**
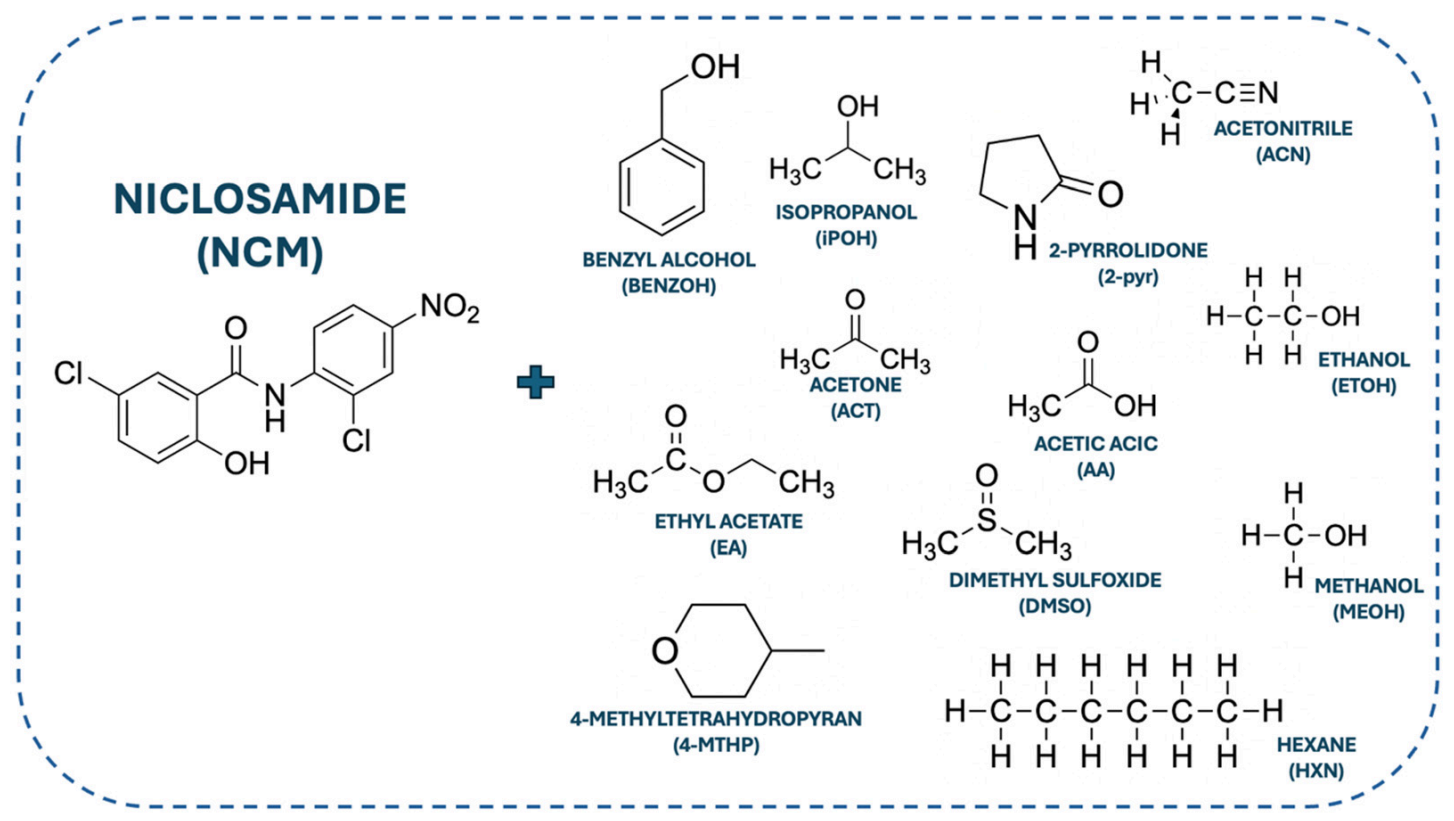
Niclosamide (NCM), dimethyl sulfoxide (DMSO), 2-pyrrolidone (2-PYR), acetic acid (AA), benzyl alcohol (BENZOH), acetone (ACT), methanol (MeOH), acetonitrile (ACN), ethanol (EtOH), ethyl acetate (EA), isopropanol (iPOH), 4-methyltetrahydropyran (4-MTHP), and hexane (HXN) molecular structures.

Niclosamide (NCM) (Figure 1), a salicylanilide derivative first introduced in the 1950s as an anthelmintic agent for the treatment of tapeworm infections, has recently emerged as a promising candidate for drug repositioning across a broad range of therapeutic areas. Initially employed for its antiparasitic activity, NCM has attracted renewed interest owing to its potential applications in antiviral, antibacterial, anticancer, and anti-inflammatory therapies. This broad pharmacological profile highlights its potential to address unmet medical needs beyond its original use in parasitology [1,2,3,4].

Despite its considerable therapeutic potential, the clinical development of NCM has been significantly hindered by its poor aqueous solubility and complex solid-state behavior. A major challenge arises from its pronounced tendency to convert easily into a highly insoluble monohydrate form (Cambridge Structural Database (CSD) refcode OBEQAN01 [5], refinement OBEQAN02 [6]). This transformation markedly reduces bioavailability, representing a critical limitation for its clinical application [7,8].

Solvates, formed through interactions between active pharmaceutical ingredients (APIs) and solvent molecules [9], can prevent or delay conversion to the monohydrate form, thereby improving both solubility and stability in pharmaceutical formulations [10]. In addition to enhancing apparent solubility, solvate formation can significantly influence the physicochemical properties and stability profile of a drug, both essential factors for successful formulation development [9,10,11].

The strong propensity of NCM to form solvates and hydrates, rather than anhydrous forms, is well documented in the literature [5,8,12,13,14]. Although the molecule exhibits limited conformational flexibility, NCM readily establishes strong intermolecular interactions that favor the formation of multicomponent solid forms. This behavior is largely attributable to its planar molecular structure, which promotes efficient molecular packing and facilitates the incorporation of solvent molecules into the crystal lattice.

To date, two monohydrates (H_A_ [5,6] and H_B_ [8]) and six solvates (with ethanol, methanol, acetone, acetonitrile, dimethyl sulfoxide and tetrahydrofuran [5,14,15,16]) have been reported. These structures are typically stabilized by π-π stacking interactions between the aromatic rings of NCM molecules. The planar architecture enables efficient stacking, generating voids or channels within the crystal lattice that can accommodate solvent molecules. The π-π interactions between parallel aromatic systems further stabilize the structure by maximizing attractive interactions between π-electron clouds [12,15].

Recent work by Kuri and co-workers has further emphasized the importance of hydrogen bonding in this context. Their studies indicate that although both anhydrous and solvated forms are stabilized by hydrogen bonds, those present in solvated structures are significantly stronger, thereby favoring solvate formation over anhydrous phases [16].

Given the marked tendency of NCM to form solvates and the potential advantages associated with these solid forms, this study aimed at identifying additional NCM solvates. An experimental screening was conducted using twelve solvents under mechanochemical conditions: dimethyl sulfoxide (DMSO), 2-pyrrolidone (2-PYR), acetic acid (AA), benzyl alcohol (BENZOH), acetone (ACT), methanol (MeOH), acetonitrile (ACN), ethanol (EtOH), ethyl acetate (EA), isopropanol (iPOH), 4-methyltetrahydropyran (4-MTHP), and hexane (HXN) (Figure 1). The solvent panel was selected to include solvents commonly employed in pharmaceutical research and processing, spanning a broad range of polarity and hydrogen-bonding ability, with the aim of maximizing the probability of identifying new crystalline solvates.

For comparison, slurry-mediated experiments were also conducted.

The products obtained through mechanochemical synthesis were comprehensively characterized using a range of solid-state analytical techniques, including powder X-ray diffraction (PXRD), differential scanning calorimetry (DSC), thermogravimetric analysis (TGA), hot-stage microscopy (HSM), Fourier-transform infrared (FT-IR) spectroscopy, and scanning electron microscopy (SEM). The crystal structure of the newly prepared NCM–2-PYR solvate was determined from PXRD data and subsequently validated by density functional theory (DFT) calculations. Furthermore, physical stability studies under various environmental conditions, together with kinetic solubility assessments, were performed to evaluate the potential suitability of this solid form for the future pharmaceutical development of NCM-based formulations.

## 2. Materials and Methods

### 2.1. Materials

NCM (5-chloro-N-(2-chloro-4-nitrophenyl)-2-hydroxybenzamide) was purchased from Sigma-Aldrich (St. Louis, MO, USA) with a declared purity of 98-101%. Acetic acid (AA) and 4-methylthetraydropyran (4-MTHP) were provided by Carlo Erba (Rodano-Milan, Italy), while hexane (HXN), methanol (MeOH), 2-pyrrolidone (2-PYR), dimethyl sulfoxide (DMSO) and isopropanol (iPOH) were purchased from Sigma-Aldrich (St. Louis, MO, USA). Ethanol (EtOH) was provided from Honeywell Riedel-de Haën (St. Louis, MO, USA), acetonitrile (ACN) from Merck KGaA, (Darmstadt, Germany), ethyl acetate (EA) from Normapur (Fontenay sous Bois, France), acetone (ACT) from Galeno (Comeana, Prato, Italy) and benzyl alcohol (BENZOH) from A.C.E.F. (Fiorenzuola D’Arda, Italy). All the active ingredients and chemicals were used without further purification.

### 2.2. Methods

#### 2.2.1. Sample Preparation

##### Mechanochemical Synthesis

Mechanochemical synthesis of NCM solvates was carried out at ambient laboratory temperature using a Retsch MM400 vibrational mill (Retsch GmbH, Haan, Germany) equipped with two 25 mL stainless steel jars, each containing a single 10 mm Ø bead. All experiments were performed under standardized conditions: a milling frequency of 25 Hz, a fixed milling time of 60 min and a constant jar filling volume corresponding to a total powder mass of 400 mg per 25 mL jar.

NCM was milled in the presence of twelve different solvents using two NCM-to-solvent molar ratios, namely 1:1 and 1:2.

##### Slurry Bridging Experiments

For comparison, slurry experiments were performed following the procedure described in a previous study [17]. Briefly, 300 mg of NCM and 5 mL of each of the twelve selected solvents was placed in 10 mL glass vials. The vials were sealed with screw caps and further secured with parafilm to minimize solvent evaporation. All suspensions were prepared in duplicate and continuously stirred at room temperature (20 °C) for seven days. After equilibration, the remaining solid phases were isolated by vacuum filtration using paper filters and subsequently analyzed by PXRD.

#### 2.2.2. Characterization Analyses

##### Laboratory PXRD Analysis

PXRD analysis was performed using a Bruker D2 Phaser benchtop diffractometer (Bruker, Manheim, Germany) operating in Bragg-Brentano geometry with Cu-Kα radiation (λ = 1.5418 Å) from a 300W low-power X-ray source (30 kV, 10 mA). Data were collected over a 2θ range of 3–40°, with a step size of 0.02° and a scan speed of 0.6°/s. Each sample was prepared by carefully pressing around 200 mg of the ground product into a steel sample holder fitted with a cylindrical polyvinylidene fluoride (PVDF) reducer.

##### DSC Analysis

For DSC analysis, samples weighing 2–4 mg were placed in 40 μL aluminum crucibles that were sealed and pierced. The analyses were performed using a Mettler Toledo DSC 3 Star System (Milan, Italy) with a heating rate of 10 °C/min from 30 to 250 °C, under a nitrogen atmosphere with a flow rate of 50 mL/min.

##### TGA Analysis

Thermogravimetric analysis (TGA) was carried out using a Mettler Toledo TGA TDA851 instrument (Milan, Italy). Approximately 5–12 mg of the powdered sample were placed in 100 μL platinum crucibles and subjected to a heating program from 30 to 250 °C at a rate of 10 °C/min, under a nitrogen atmosphere with a flow rate of 50 mL/min.

##### HSM Analysis

A Leica optical microscope with 10× magnification was used alongside a Mettler FP5 hot-stage microscope to visually monitor the sample during heating. Images were captured through the microscope’s integrated camera and viewed using Webcam Companion software. The heating conditions for the samples were the same as those used in the DSC and TGA analyses.

##### (FT-IR)-ATR Analysis

Infrared spectra using Attenuated Total Reflectance (ATR) were acquired with a Shimadzu IRAffinity-1S FT-IR spectrometer (Shimadzu Corporation, Kyoto, Japan). Measurements were conducted over a range of 400–4000 cm^−1^ with a resolution of 4 cm^−1^ and involved 20 scans.

##### SEM Analysis

SEM images of NCM–2-PYR were acquired by placing the powdered sample on aluminum stubs coated with double-sided carbon tape, followed by gold sputter-coating using a K550X sputter coater (Emitech, Quorum Technologies Ltd., East Sussex, UK). The samples were then examined with a scanning electron microscope (Quanta 250, FEI, Hillsboro, OR, USA) equipped with a secondary electron detector. A working distance of 10 mm was used to achieve the desired magnifications, and the accelerating voltage was set at 30 kV.

##### Synchrotron X-Ray Diffraction and Solution of the Crystal Structure

The data collection of the solvate obtained in powder form was performed at the X-ray diffraction beamline (XRD2) of Elettra Synchrotron (Trieste, Italy) [18]. The sample was measured at 298 K in transmission mode filling a boron capillary of 0.5 mm of diameter. The powder data was acquired using a monochromatic wavelength of 1.00 Å on Pilatus 6M hybrid-pixel area detectors (DECTRIS Ltd., Baden-Daettwil, Switzerland). The two-dimensional powder pattern was integrated using GSAS II software [19], after preliminary calibration of hardware setup, using a capillary filled with LaB_6_ standard reference powder (NIST 660a). The indexing of the powder pattern and the structure solution were performed using the program EXPO2014 through a simulated annealing (SA) protocol [20]. The starting structural model containing two NCM molecules and two molecules of 2-pyrrolidone were built using Material Studio software v 7.0. (MS) [21] and then geometrically optimized by the Dmol3 code implemented in MS. The Rietveld refinement on the structure obtained by the SA was then performed using GSAS II software.

##### DFT Calculations

The solved refined structure was then subjected to geometry optimization by DFT periodic calculations, as provided by the BAND engine of the AMS program [21], using a Triple-ζ plus Polarization (TZP) Slater Type Orbital (STO) basis set and the GGA PBE functional [22]. Semi-empirical D3BJ dispersion corrections [23,24] were included and the experimental unit-cell parameters were kept fixed during geometry optimization.

##### Kinetic Solubility Studies

The solubility of the newly prepared NCM–2-PYR solvate, in comparison with anhydrous NCM Form A, was evaluated in screw-capped, 20 mL glass vials by adding an excess of powder to 17 mL of distilled water. Each experiment was performed in a thermostatic chamber maintained at 37 ± 0.5 °C, with the sample mounted on a vertically rotating plate to ensure continuous agitation throughout the experiment.

Kinetic solubility studies were carried out over 10 h to monitor dissolution behavior over time. Aliquots were withdrawn at predetermined time intervals (1, 2, 3, 4.5, 6, 7.5, and 10 h), filtered through a 0.45 μm regenerated cellulose (RC) syringe membrane filter (Minisart, Sartorius, Goettingen, Germany), and analyzed.

For equilibrium solubility determination, samples were collected after 48 h and 72 h and filtered under the same conditions.

In parallel, the solid residues recovered at selected time points were collected, dried, and analyzed by PXRD to assess possible solid-state transformations during the experiments. The concentration of dissolved NCM was determined spectrophotometrically (Agilent 8453 UV–Vis spectrophotometer, Hewlett Packard, Wilmington, Germany) at 333 nm.

All measurements were carried out in triplicate for each sample and time point.

#### 2.2.3. Physical Stability Assessment of NCM–2-PYR Under Different Stress Conditions

##### Physical Stability in the Solid-State over Time

The solid-state stability of NCM–2-PYR was evaluated at room temperature (20 °C) by storing the sample in a desiccator over calcium chloride for six months. Periodic monitoring, as well as a final assessment at the end of the storage period, was carried out by PXRD under the conditions described in the experimental section. Section 2.2.3.

##### Resistance to Compression

To investigate the effect of mechanical stress, the new NCM solvate was subjected to compression at 10 Ton for 3 min. This evaluation is particularly relevant because solvates often display limited resistance to compression [25] and NCM is primarily formulated as oral tablets [7].

##### Resistance to Conversion into NCM H_A_ Under High Relative Humidity (RH)

To evaluate whether NCM–2-PYR can prevent the formation of the insoluble NCM monohydrate H_A_—which typically develops within one month under ambient humidity conditions [5,7,8]—the stability of this multicomponent system was investigated under high relative humidity (RH). Samples were stored in a sealed chamber containing a saturated aqueous NaCl solution, which maintains an RH of 75% at room temperature [26]. Structural stability was monitored by PXRD after 24, 48, and 144 h of exposure to detect any phase transformations or the onset of the monohydrate formation.

## 3. Results

### 3.1. Screening of NCM Solvates—Introduction

A crystal form screening of NCM was performed using liquid-assisted grinding (LAG) and, for comparison, slurry-bridging experiments starting from pure anhydrous NCM Form A (CSD refcode HEBFUR [27]). Twelve commonly used solvents were investigated at two solid-to-solvent molar ratios (1:1, 1:2). The selected solvents and the outcomes of both LAG and slurry experiments are summarized in Table 1.

LAG experiments with EtOH, EA, 4-MTHP, iPOH, and HXN resulted in the formation of a mixture of anhydrous NCM Form A (CSD refcode HEBFUR [28]) and NCM monohydrate H_A_ (CSD refcode OBEQAN02 [5,6]). The same mixture was also obtained under slurry conditions using ACN, ACT, and HXN.

LAG treatment in the presence of AA, ACT, and BENZOH led to the formation of the H_B_ monohydrate (CSD refcode OBEQAN [8,28,29]), reported in the literature as the thermodynamically most stable monohydrate form. Under slurry conditions, the same H_B_ phase was observed only in the presence of BENZOH, whereas slurry experiments with 4-MTHP, iPOH, and AA resulted in the recovery of the starting anhydrous NCM Form A.

Slurry experiments performed with EtOH and EA yielded NCM polymorph B (CSD refcode HEBFUR01 [5]).

LAG experiments with ACN produced a mixture of the NCM–ACN monosolvate [5] and NCM H_A_.

Experiments conducted with MeOH showed distinct outcomes depending on the applied method: LAG led to the formation of the NCM–MeOH monosolvate (CSD refcode GOJLIC [14,15]), whereas slurry experiments produced a mixture of the NCM–MeOH monosolvate and NCM H_A_.

The NCM–DMSO solvate corresponded to the structure recently reported by Kuri et al. [16] and deposited in the CSD under the refcode WOXVIS. Notably, in the present study, the DMSO monosolvate was obtained by LAG within 1 h, significantly reducing the preparation time compared to the two-day slow solvent evaporation previously described [16].

By contrast, the NCM–2-PYR solvate is novel and, to the best of our knowledge, has not been previously reported. In both LAG and slurry experiments, the formation of this solvate was reproducible and resulted in complete conversion to a single crystalline phase, as confirmed by PXRD analysis. For this reason, it was subjected to extensive characterization, including crystal structure solution, as described in the following section.

**Figure 2:**
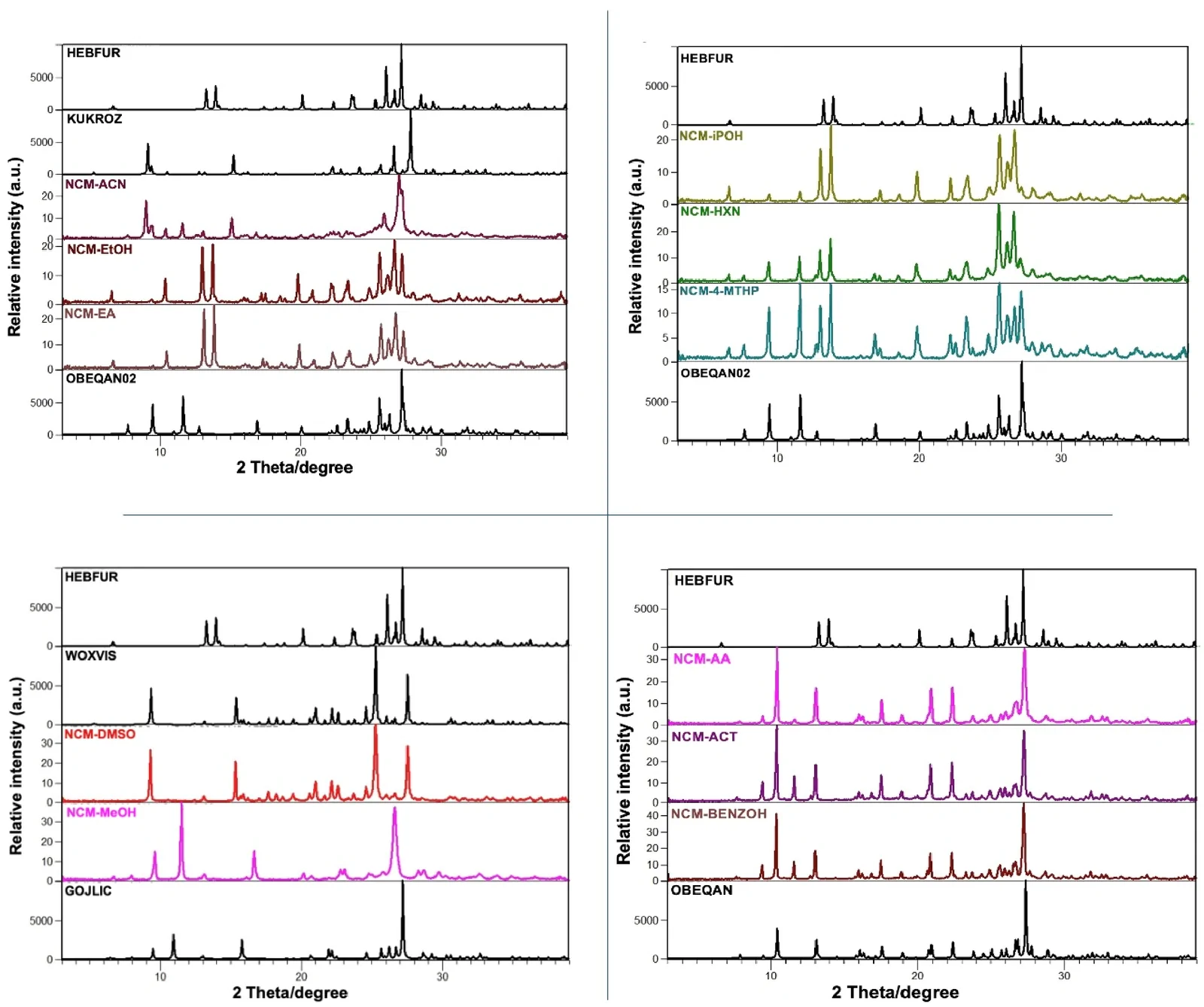
PXRD patterns of samples obtained by LAG in the presence of eleven of the selected solvents (ACN violet, EtOH brown, EA light brown, iPOH olive, HXN green, 4-MTHP light green, DMSO red, MeOH pink, AA pink, ACT violet, BENZOH brown), compared with reference patterns of known NCM solid forms: NCM Form A (CSD refcode HEBFUR, black), NCM Form B (CSD refcode HEBFUR01, black), NCM monohydrate H_A_ (CSD refcode OBEQAN02, black), NCM monohydrate H_B_ (CSD refcode OBEQAN, black), NCM–ACN monosolvate (CSD refcode KUKROZ, black), NCM–MeOH monosolvate (CSD refcode GOJLIC, black), and NCM–DMSO monosolvate (CSD refcode WOXVIS, black).

**Figure 3:**
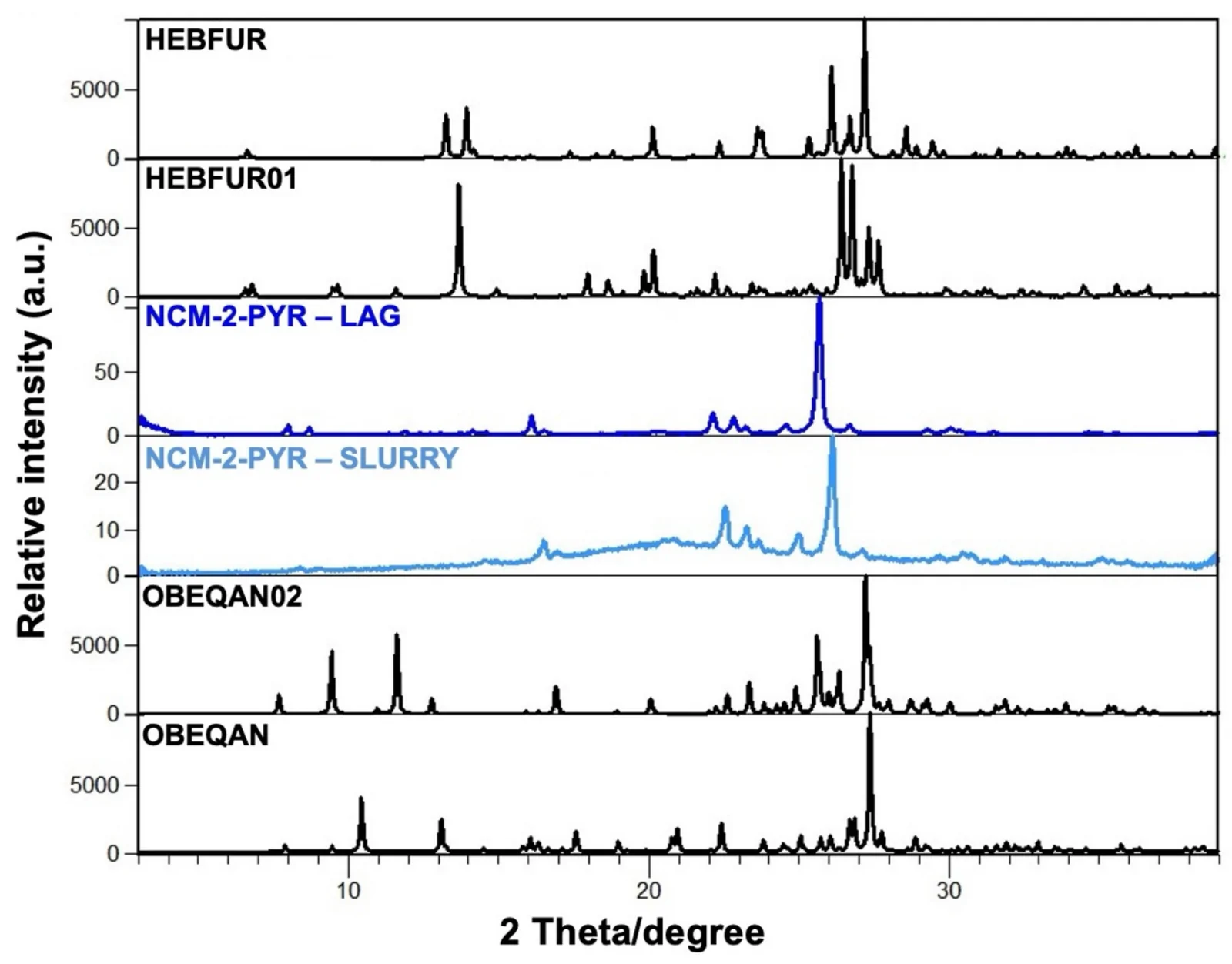
PXRD of the new phase NCM–2-PYR obtained via LAG (blue) and via slurry (light blue) compared with reference patterns of known NCM solid forms: NCM Form A (CSD refcode HEBFUR), NCM Form B (CSD refcode HEBFUR01), NCM monohydrate H_A_ (CSD refcode OBEQAN02), NCM monohydrate H_B_ (CSD refcode OBEQAN).

PXRD patterns obtained from LAG experiments with eleven of the selected solvents are shown in Figure 2 and compared with reference patterns of known NCM solid forms. The PXRD pattern of the NCM–2-PYR system is presented separately in Figure 3 together with its structural characterization. The corresponding PXRD data for the slurry experiments are provided in the Supplementary Information (Figures S1–S4).

#### 3.1.1. NCM-2-PYR Solvate

The NCM–2-PYR solid was obtained as a straw-yellow crystalline powder. Its PXRD pattern (Figure 3) is clearly distinguishable from those of previously reported NCM solid forms and is characterized by a prominent reflection at approximately 25.9° 2θ. The absence of reflections attributable to the starting materials indicates that the mechanochemical synthesis proceeds with high conversion.

Figure 3 also shows the PXRD pattern of the sample obtained via the slurry method. Although both patterns correspond to the same crystalline phase, minor differences in peak intensities are observed, likely reflecting differences in crystallinity and particle morphology arising from the two preparation methods.

Comparison with the slurry approach further highlights the practical advantages of the mechanochemical route under the investigated conditions. In addition to the significantly reduced processing time (1 h vs. 7 days) and the limited amount of solvent required, mechanochemistry is well recognized as an effective approach for accessing kinetically favored solid forms that may be difficult to obtain under solution-mediated conditions, which generally probe transformations closer to thermodynamic equilibrium. In the present case, both approaches yielded the same crystalline phase; however, the material obtained by LAG was recovered as a free-flowing powder, whereas the slurry product exhibited a highly pasty consistency, making its isolation and handling considerably more challenging without additional processing. These features further support the suitability of mechanochemistry as a rapid and solvent-efficient approach for exploratory solid-form screening.

**Figure 4:**
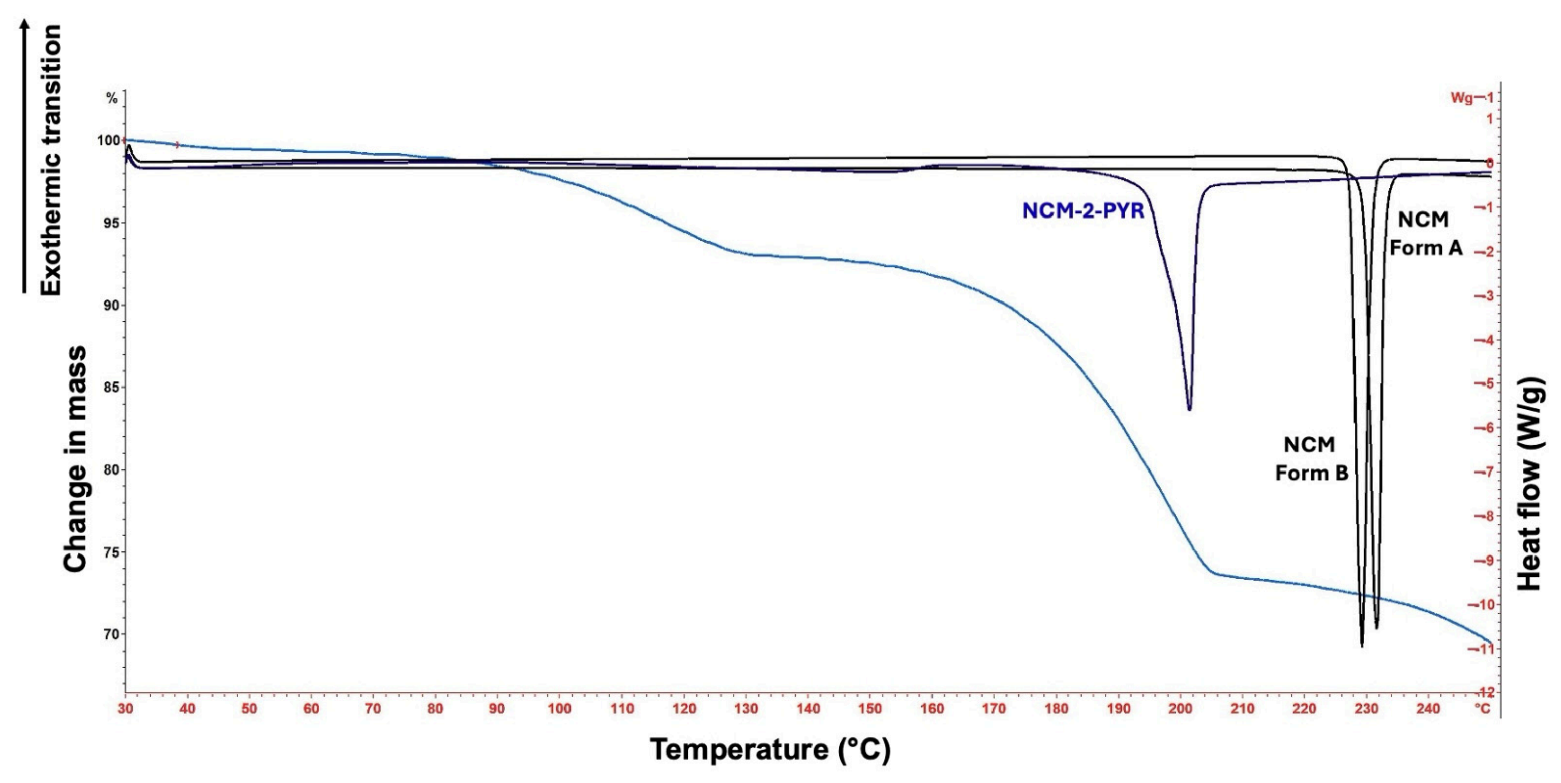
TGA curve (light blue) and DSC traces of the new phase NCM–2-PYR (blue), compared to NCM Form A (black) and NCM Form B (black).

Figure 4 shows the DSC (blue) and TGA (light blue) curves of NCM–2-PYR.

For NCM–2-PYR, the DSC trace is dominated by a distinctive endothermic event at 200.97 °C with an enthalpy of 113.57 J g^−1^, attributable to the collapse of the solvated crystal lattice. This thermal event, characteristic of the 2-PYR solvate, occurs at a temperature markedly different from that of the parent NCM Form A, and no melting event corresponding to either of the anhydrous forms of NCM is observed.

Comparison with the TGA profile indicates that the mass loss associated with desolvation overlaps with the thermal event observed in the DSC curve. An experimental mass loss of 19.87% was observed, in excellent agreement with the theoretical value expected for a 1:1 NCM–2-PYR stoichiometry (20.64%). The weight loss, consistent with an equimolar NCM-to-2-PYR stoichiometry, occurs over a relatively broad temperature range. This gradual solvent release accounts for the broad and low-intensity endothermic signal observed in the DSC trace prior to collapse of the crystal lattice. The shape of this shallow endotherm is characteristic of 2-PYR solvates and closely resembles that reported for solvates of theophylline [17,30].

To further elucidate this peculiar thermal behavior and verify whether desolvation and lattice collapse occur concomitantly at approximately 200 °C, the sample was heated stepwise (first to 185 °C and then to 225 °C) and subsequently analyzed by PXRD (Figure S5). In the first case, the diffraction pattern remained identical to that of the initial solvate. Contrarily, the sample heated to 225 °C exhibited a different PXRD pattern, with extra peaks corresponding to anhydrous NCM.

HSM (Figure S6) does not provide additional insights into the early thermal events, as only the collapse of the solid at approximately 200 °C can be clearly observed, while any preceding transformations cannot be distinguished. However, observation of the same sample during cooling (from 210 °C to ambient temperature) reveals that, following collapse of the solvated solid, crystalline anhydrous NCM forms with a characteristic needle-like habit, while liquid 2-PYR appears as droplets on the slide.

During the first heating stage, after NCM–2-PYR collapse, NCM does not recrystallize and therefore its melting is not detected in the DSC trace extended to 250 °C. In contrast, repeated heating–cooling–heating cycles demonstrate that, once the solvated phase has collapsed, crystalline anhydrous NCM is formed. PXRD analysis of the recovered material confirms that this phase corresponds to NCM Form A (Figure S7).

**Figure 5:**
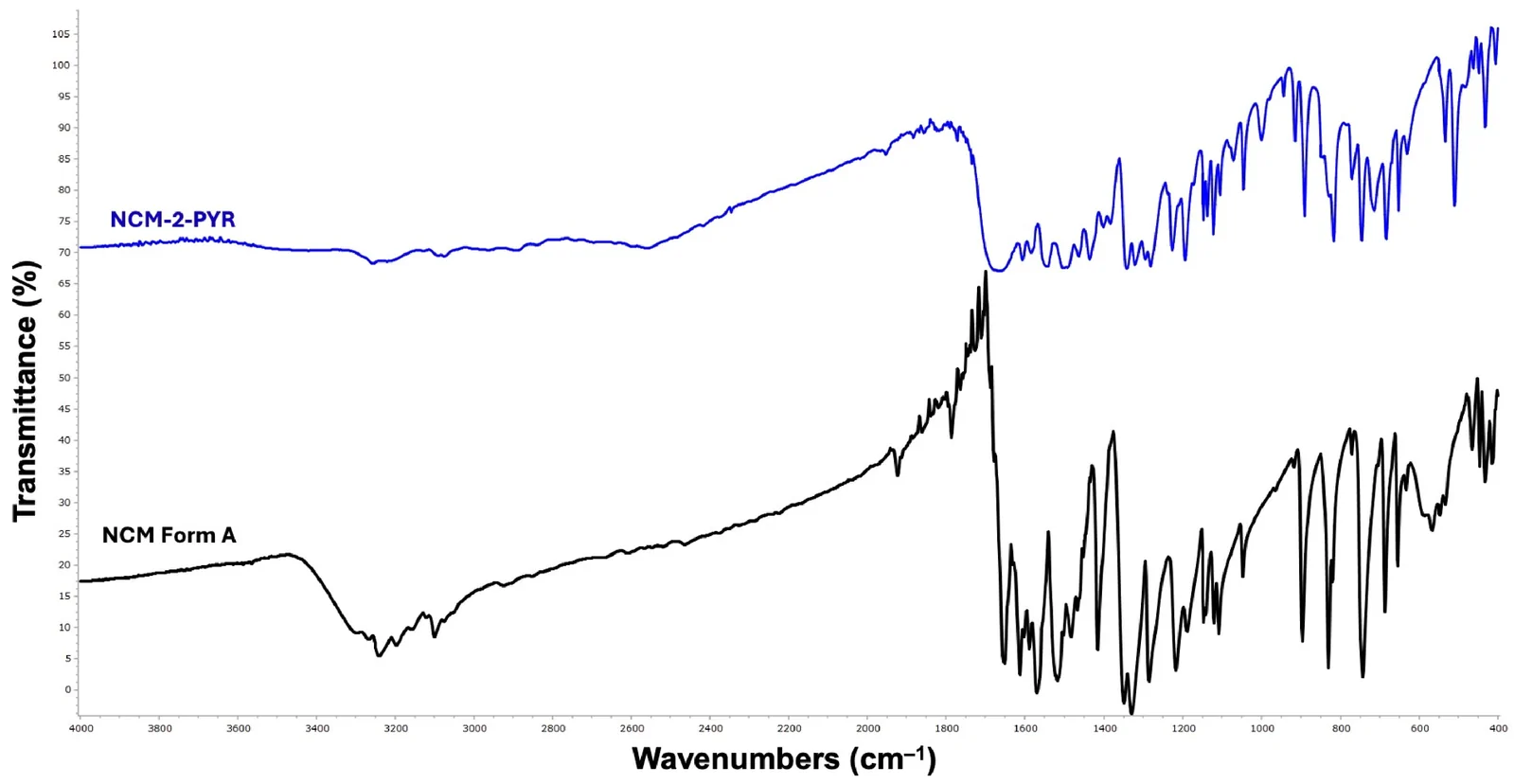
FT-IR ATR spectra of NCM–2-PYR (blue) and anhydrous NCM Form A (black).

The FT-IR ATR spectrum of the NCM–2-PYR solvate (Figure 5) differs markedly from that of anhydrous NCM Form A, confirming the formation of a new solid form of NCM.

Figure 5 compares the spectrum of the NCM–2-PYR solvate with that of anhydrous NCM Form A, allowing identification of the functional groups of NCM involved in interactions with 2-PYR. Attention was devoted to the spectral regions 3500–3300 cm^−1^ (–NH and –OH stretching) and 1700–1600 cm^−1^ (C=O stretching).

The analysis revealed the following:

- The absence of the doublet at 3576 and 3491 cm^−1^, associated with OH stretching of NCM [31], suggests that the phenolic –OH group participates in hydrogen bonding with the carbonyl group of 2-PYR.
- The amine –NH group of NCM also appears to be involved in the interaction, as the bands corresponding to its stretching (3237.8 and 3194.4 cm^−1^ [32]) and bending (897 cm^−1^ [33]) modes are shifted in the NCM–2-PYR spectrum to 3257, 3214, and 890 cm^−1^, respectively.
- Upon solvation with 2-PYR, the NO_2_ stretching band of NCM (around 1520 cm^−1^) remains detectable but shows slight shifts and noticeable band broadening. In addition, band splitting is observed, suggesting that the two nitro groups experience different local environments within the solvated structure.
- The ν(C=O) band of 2-PYR, observed at approximately 1680 cm^−1^ in the neat solvent [33], is shifted to lower wavenumbers in the solvate, consistent with hydrogen-bond formation with NCM.
- Significant differences are also observed in the 1700–1200 cm^−1^ region, which displays a spectral pattern distinct from that of anhydrous NCM, further supporting the formation of a new solid phase.

SEM analysis showed that the newly obtained 2-PYR monosolvate consists of agglomerates of fine particles with a relatively uniform overall appearance. Although particle agglomeration prevents a detailed assessment of the morphology of individual crystallites, the sample clearly exhibits a morphology distinct from that of the starting NCM Form A. No residual particles attributable to the starting material were observed.

**Figure 6:**
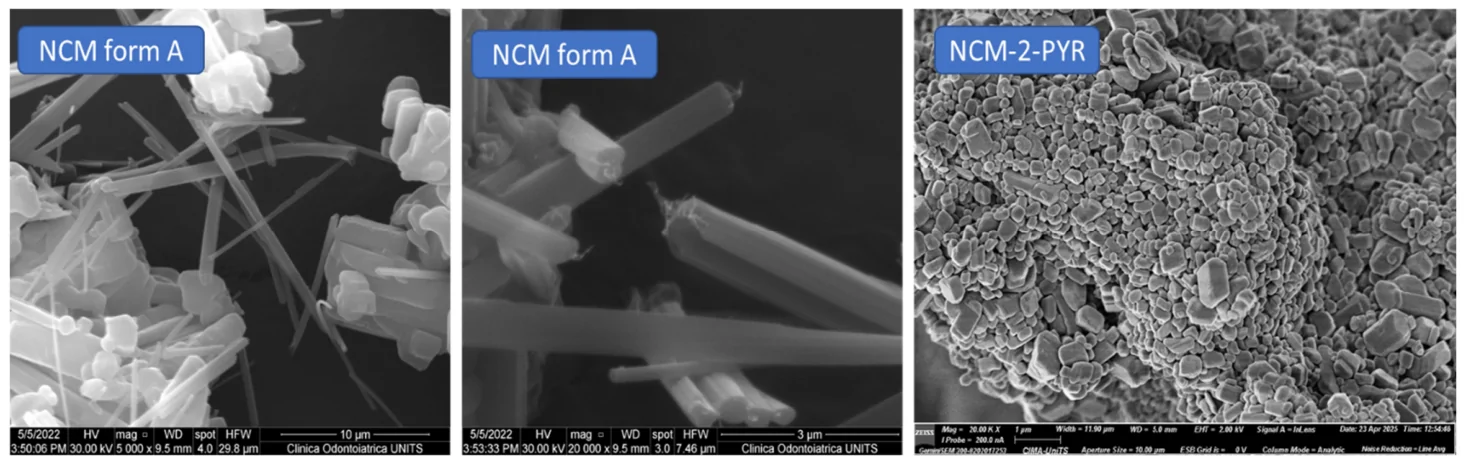
SEM images of the starting NCM Form A (5000×, **left**; 20,000×, **center**) and the NCM–2-PYR monosolvate (20,000×, **right**).

In contrast, as also visible in Figure 6, NCM Form A displays a markedly different morphology, consisting predominantly of rectangular particles with protrusions, as previously reported by Van Tonder et al. [8], together with some needle-shaped crystals.

**Figure 7:**
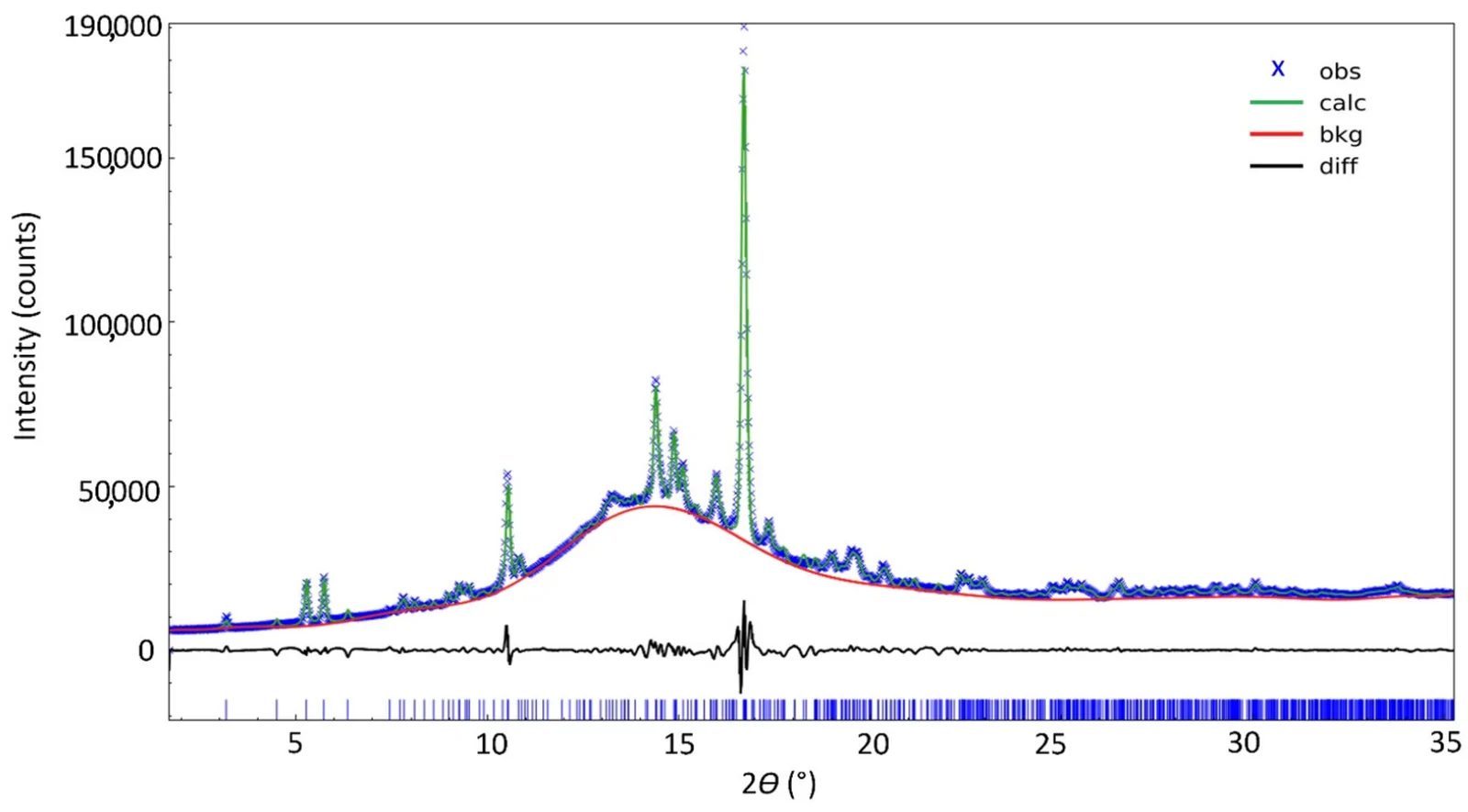
Rietveld refinement profile fit (obtained after the simulated annealing with EXPO14) of the solvate: in blue crosses the experimental pattern (recorded using Synchrotron radiation, wavelength: 1.00 Å), in green the calculated one. The residuals are displayed on the bottom in black and the reflection ticks in blue.

**Figure 8:**
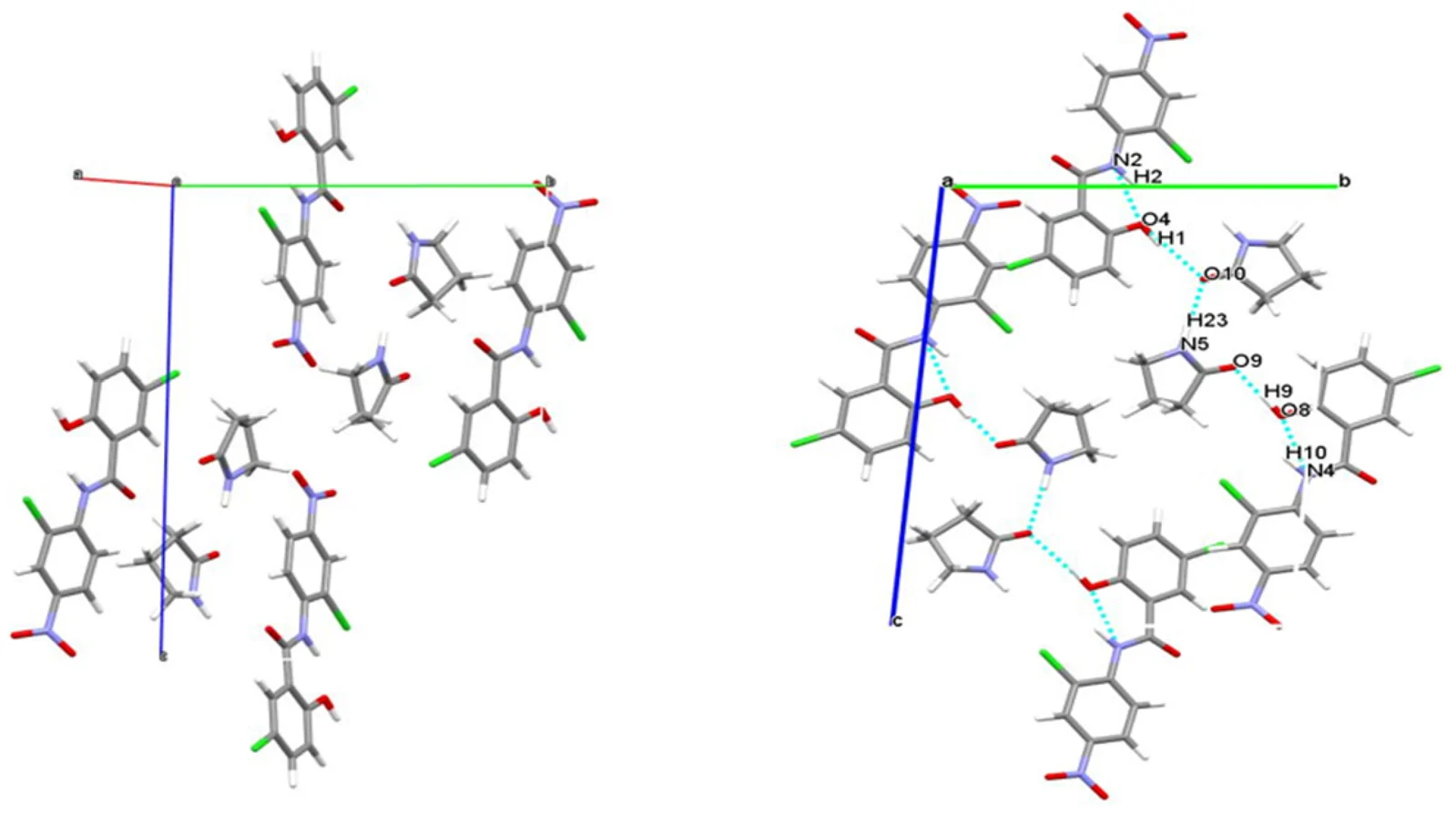
a. (**left**) Capped-stick representation of the proposed structure of NCM–2-PYR viewed along the a-axis; b. (**right**) H bond between the molecules shown as dashed cyan lines.

Two separate simulated annealing runs were performed: the first maintaining a rigid planar geometry for the NCM molecules, and the second allowing the rotation of the terminal nitro groups. Although there are two crystallographically independent molecules in the asymmetric unit (ASU), only one exhibited a significant rotation of these groups (157.1°). This flexible approach led to a substantial improvement in the agreement between the experimental and calculated patterns, so this structure was selected and used for the Rietveld refinement (Figure 7). The crystal structure of NCM–2-PYR is presented in Figure 8. The samples resulted in a *P*-1 triclinic unit cell with the following parameters: a 7.619147 (1) Å, b 12.943071 (5) Å, c = 18.403814 (5) Å, α = 93.7942 (8)°, β = 99.3386 (1)°, γ 97.5293 (1) density = 1.55g/cm^3^ and volume 1768.295 (3) Å^3^. The number of formula units (2NCM:2 2-PYR) per unit cell is Z = 4, containing two independent molecules of NCM and 2 independent molecules of 2-PYR. A first whole powder pattern fitting (Pawley method) on the experimental powder pattern resulted in a good Rwp of 1.68%. The final Rietveld refinement was subsequently carried out using GSAS-II, applying soft restraints on bond distances (±0.03 Å) and bond angles (±0.1°) to maintain chemically reasonable molecular geometries. The refinement converged with Rp = 8.70%, Rwp = 15.47%, Rexp = 7.00%, GOF = 2.20, and an RBragg factor of 7.48%, confirming the reliability of the proposed structural model. CCDC 2561439 contains the supplementary crystallographic data for NCM–2-PYR solvate. These data can be obtained free of charge from The Cambridge Crystallographic Data Centre via https://www.ccdc.cam.ac.uk/structures (accessed on 11 June 2026).

The crystal packing is governed by a network of hydrogen bonds (Figure 8, right). Notably, intermolecular O4–H1⋯O10 and O8–H9⋯O9 interactions link NCM and 2-PYR molecules, while the N2–H2⋯O4 and N4–H10⋯O8 interactions, already present in pure NCM, are retained. A strong N5–H23⋯O10 hydrogen bond is also observed between 2-PYR units.

To further validate the structure solution obtained from powder diffraction data, the refined model was subjected to periodic DFT geometry optimization using a fixed-cell approach, in which only atomic coordinates were allowed to relax while the experimental unit-cell parameters were kept constant. The optimized structure remained in good agreement with the experimental model, showing only limited atomic displacements and preserving the overall molecular packing arrangement. Importantly, the hydrogen-bond network identified experimentally was retained after optimization, including the intermolecular interactions between NCM and 2-PYR molecules as well as the intramolecular N–H⋯O motifs characteristic of NCM. These results support the reliability of the structure solution obtained from PXRD data and indicate that the proposed packing corresponds to a stable structural arrangement.

**Figure 9:**
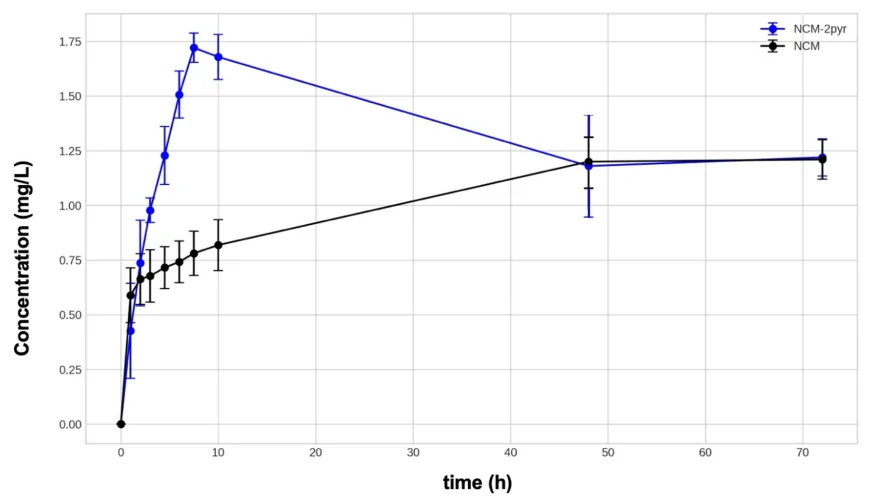
Kinetic and equilibrium solubility profiles of NCM–2-PYR (blue) and anhydrous NCM Form A (black). Data are reported as mean ± standard deviation (n = 3).

Solubilization kinetics studies of the 2-PYR solvate and NCM Form A were carried out over 72 h at 37 °C, with frequent sampling during the first 10 h followed by one sampling point per day (Figure 9).

NCM Form A showed a slow increase in dissolved concentration, reaching a plateau after 48 h with a value of 1.20 ± 0.23 mg/L, which remained unchanged up to 72 h. PXRD analysis revealed that already after the first sampling point, NCM was almost completely converted into NCM monohydrate H_A_.

In contrast, NCM–2-PYR displayed a markedly different behavior, characterized by a progressive increase in dissolved concentration up to a maximum at approximately 10 h, followed by a decrease until reaching the same concentration value as NCM Form A. The dissolved concentration of NCM–2-PYR was significantly higher than that of Form A between 2 and 10 h of analysis, whereas from 48 h onward the two profiles became comparable. Unlike NCM Form A, NCM–2-PYR had not yet reached equilibrium after 48 h, as indicated by the high standard deviation. Equilibrium was achieved only after 72 h (1.21 ± 0.084 mg/L), with a concentration value comparable to that of commercial NCM.

The explanation for these solubilization kinetics comes from PXRD analysis of the solids collected at the different sampling times. Pristine NCM rapidly converted, already after the first sampling point, almost completely into NCM monohydrate H_A_ (Figure S8). In the case of the 2-PYR solvate, however, conversion into NCM monohydrate was observed only after approximately 10 h and remained only partial (NCM–2-PYR + H_A_), thus accounting for the subsequent decrease in dissolved concentration (Figure S9). The delayed equilibrium reached only after 72 h is also consistent with the slow conversion of the solvated solid into the monohydrate phase (Figures S10 and S11). These results demonstrate that NCM–2-PYR delays the solid-state transformation in water to the poorly soluble monohydrate H_A_ compared to anhydrous NCM, resulting in a transient improvement in dissolution behavior under physiologically relevant conditions.

Although the increase in dissolved concentration is relatively modest and not comparable to the much larger solubility enhancements typically achieved with amorphous solid dispersions, the improved dissolution performance of NCM–2-PYR arises from its delayed hydration combined with its excellent solid-state stability, highlighting the different pharmaceutical role of this crystalline solvate.

#### 3.1.2. Stability Assessment of NCM–2-PYR Under Different Stress Conditions

The solid-state stability of the NCM–2-PYR monosolvate was assessed at room temperature (20 °C) by storing samples obtained from different batches in a desiccator over calcium chloride for 18 months. Periodic analyses, together with a final evaluation of the stored samples, were carried out using PXRD.

**Figure 10:**
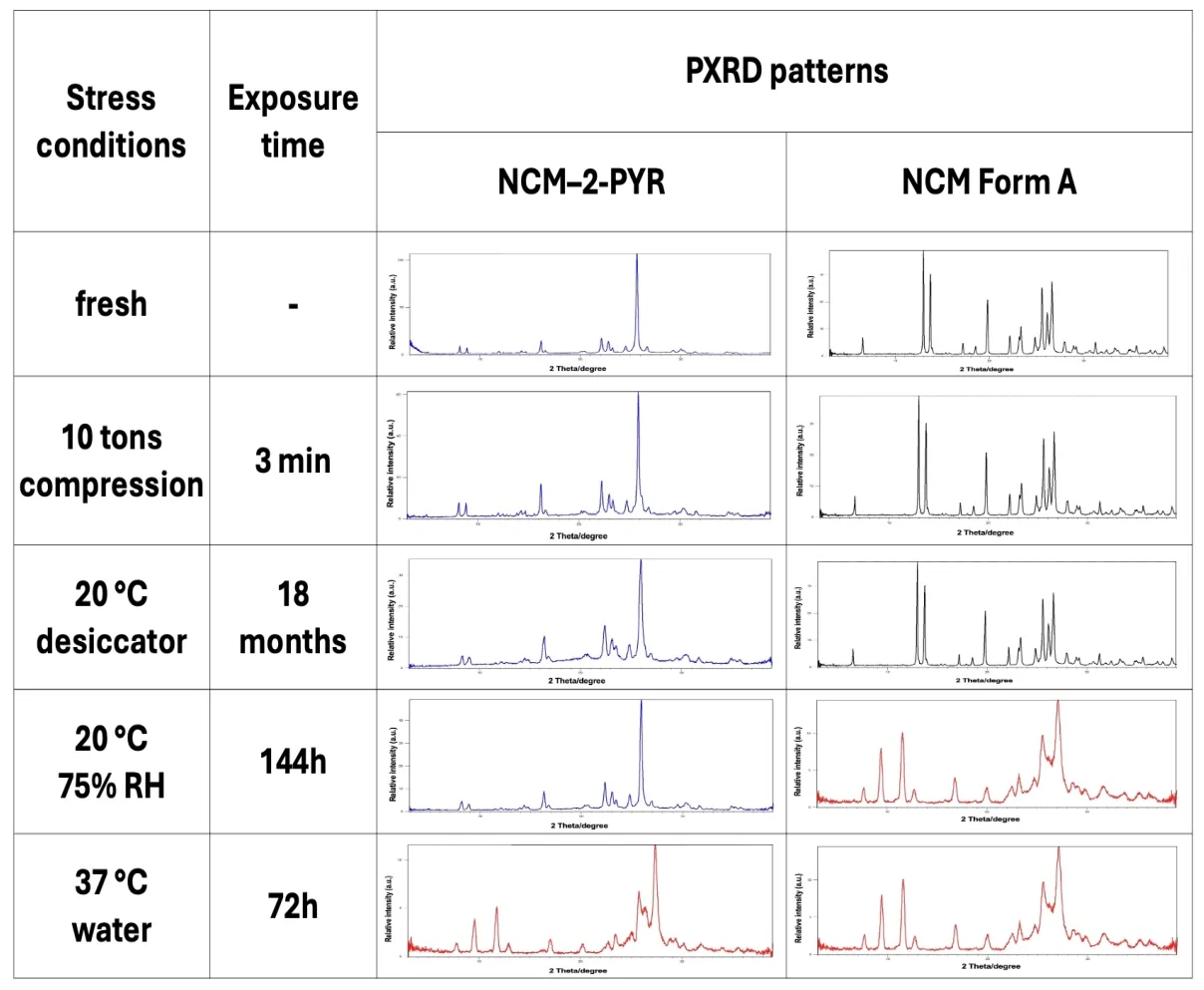
Overview of the stability study of four NCM–2-PYR samples. The summary table reports the applied stress conditions and exposure times on the left, while the corresponding sample identities and PXRD patterns are shown on the right. PXRD patterns shown in red indicate samples that converted into insoluble niclosamide monohydrate H_A_.

Several batches of monosolvate were prepared and were found to remain stable under these conditions. No differences were observed between freshly prepared materials and those stored for 18 months (Figure 10), indicating excellent long-term solid-state stability.

To evaluate the effect of mechanical stress, NCM–2-PYR monosolvate samples were compressed at 10 tons for 3 min. This test is particularly relevant because solvates often exhibit limited resistance to compression [26], while NCM is predominantly formulated as oral tablets [7]. The NCM–2-PYR monosolvate demonstrated good mechanical stability, as no changes in the PXRD patterns were observed after compression (Figure 10).

Given the satisfactory stability of the NCM–2-PYR monosolvate under the stress conditions described above, its resistance to conversion into the poorly soluble NCM monohydrate H_A_ was subsequently investigated.

The formation of NCM H_A_, which typically occurs within one month of exposure to ambient humidity, represents a major challenge due to its extremely low solubility and the well-known “cement-like” behavior of the resulting material. This property significantly reduces the dissolution rate and bioavailability of NCM, consistent with its classification as a Biopharmaceutical Classification System (BCS) class II compound, ultimately limiting its therapeutic effectiveness [5,7,8].

The aim of these experiments was therefore to assess whether the new 2-PYR monosolvate could inhibit the formation of NCM H_A_ and thereby mitigate these undesirable physicochemical properties.

Samples were stored in a sealed chamber containing a saturated aqueous NaCl solution, which maintains a relative humidity of 75% at room temperature [26]. PXRD analyses were performed after 24, 48, and 144 h of exposure to monitor possible phase transformations.

Comparison of the diffractograms recorded before and during exposure to humidity showed no evidence of NCM H_A_ formation, even after 144 h. This behavior contrasts sharply with that of anhydrous NCM Form A, which converts to the monohydrate within only a few hours under the same conditions. A comparison between NCM–2-PYR monosolvate and native NCM Form A, both as freshly prepared materials and after 144 h of exposure to high relative humidity, is presented in Figure 10.

Complementary experiments conducted in water at 37 °C for 72 h, under conditions relevant to solubility testing (see solubility tests results), demonstrated that both anhydrous NCM Form A and the NCM–2-PYR monosolvate convert to the H_A_ monohydrate.

The enhanced resistance of NCM–2-PYR toward moisture-induced conversion can be rationalized considering its crystal structure. The incorporation of 2-pyrrolidone into the crystal lattice through intermolecular hydrogen-bonding interactions contributes to the stabilization of the crystal packing, making the transformation into the H_A_ monohydrate less readily accessible under humid conditions. Although hydration ultimately occurs upon prolonged exposure to liquid water, the slower solid-state transformation is consistent with the delayed conversion observed during the kinetic solubility experiments.

All these results indicate that, although hydration cannot be prevented upon direct contact with water, the solvate exhibits enhanced resistance to moisture-induced conversion under typical storage conditions.

## 4. Conclusions

This study confirms the pronounced tendency of niclosamide to form solvated and hydrated solid forms. Mechanochemical screening, supported by slurry experiments, proved to be an efficient strategy for rapidly exploring the solid-state landscape and identifying new solvated solid forms using a limited set of strategically selected solvents. The combined use of PXRD, DSC, TGA, FT-IR, HSM, and SEM enabled a comprehensive characterization of the obtained phases and clarified their relationships with previously reported niclosamide solid forms.

Among the investigated systems, a previously unreported stoichiometric 2-pyrrolidone solvate (NCM–2-PYR) was identified. Its crystal structure was solved from powder X-ray diffraction data and supported by periodic DFT calculations, revealing a triclinic P-1 structure. The new solvate exhibits thermal behavior characterized by desolvation concomitant with crystal lattice collapse, together with a well-defined 1:1 NCM–2-PYR stoichiometry.

NCM–2-PYR remained stable for at least 18 months under dry storage conditions, showed resistance to compression, and exhibited enhanced resistance to moisture-induced conversion into the poorly soluble H_A_ monohydrate. Although hydration still occurred upon direct contact with water, kinetic solubility studies demonstrated a delayed solid-state transformation compared with anhydrous NCM Form A, resulting in higher dissolved concentrations during the initial stages of dissolution.

Overall, these findings expand the known solid-state landscape of niclosamide and demonstrate that mechanochemical screening is an effective approach for identifying previously unknown solvated forms. The physicochemical properties of the NCM–2-PYR solvate suggest that this newly identified solid form warrants further investigation in future formulation and pharmaceutical development studies.

## Figures and Tables

**Table 1:** Twelve screened solvents and observed NCM solid forms via LAG and slurry-bridging in the presence of each liquid additive (bold type indicates the unreported solvate).

Solvent	Outcomes	
	LAG	Slurry-Bridging
**ACN**	NCM-ACN monosolvate+NCM H_A_	NCM Form A + NCM H_A_
**EtOH**	NCM Form A + NCM H_A_	NCM Form B
**EA**	NCM Form A + NCM H_A_	NCM Form B
**4-MTHP**	NCM Form A + NCM H_A_	NCM Form A
**HXN**	NCM Form A + NCM H_A_	NCM Form A + NCM H_A_
**iPOH**	NCM Form A + NCM H_A_	NCM Form A
**MeOH**	NCM-MeOH monosolvate	NCM-MeOH monosolvate + NCM H_A_
**DMSO**	NCM-DMSO monosolvate	NCM-DMSO monosolvate
**2-PYR**	**NCM-2-PYR**	**NCM-2-PYR**
**AA**	NCM-H_B_	NCM Form A
**ACT**	NCM-H_B_	NCM Form A + NCM H_A_
**BENZOH**	NCM-H_B_	NCM-H_B_

## Data Availability

The original contributions presented in this study are included in the article/Supplementary Materials. Further inquiries can be directed to the corresponding author.

## Supplementary Materials

The following supporting information can be downloaded at: https://www.mdpi.com/article/10.3390/pharmaceutics18080906/s1. Figure S1. PXRD patterns of samples prepared via the slurry method in the presence of ACT, ACN and HXN, compared with the corresponding known NCM solid forms: NCM Form A (CSD refcode HEBFUR) and NCM H_A_ (CSD refcode OBEQAN02); Figure S2. PXRD patterns of samples prepared via the slurry method in the presence of EA and EtOH, compared with correlated known NCM solid forms: NCM Form A (CSD refcode HEBFUR) and NCM Form B (CSD refcode HEBFUR01); Figure S3. PXRD patterns of samples prepared via the slurry method in the presence of 4-MTHP, iPOH, AA and BENZOH, compared with correlated known NCM solid forms: NCM Form A (CSD refcode HEBFUR) and NCM H_B_ (CSD refcode OBEQAN); Figure S4. PXRD patterns of samples prepared via the slurry method in the presence of MeOH and DMSO, compared with correlated known NCM solid forms: NCM Form A (CSD refcode HEBFUR), NCM H_A_ (CSD refcode OBEQAN02), NCM– MeOH (CSD refcode GOJLIC) and NCM–DMSO monosolvate (CSD refcode WOXVIS); Figure S5. PXRD patterns of NCM–2-PYR at room temperature (blue), after heating to 185 °C (dark blue), and after heating to 225 °C (very dark blue), compared with the reference pattern of anhydrous NCM Form A (CSD refcode HEBFUR); Figure S6. Photomicrographs of NCM–2-PYR monosolvate collected during HSM experiments (magnification 10×); Figure S7. PXRD pattern of the solid recovered after the heating–cooling–heating experiment (dark blue), compared with the reference pattern of NCM Form A (CSD refcode HEBFUR); Figure S8. PXRD patterns of the solid residues ((a) NCM–2-PYR and (b) NCM) recovered after 1 h in aqueous medium during kinetic solubility experiments (red), compared with patterns of fresh NCM–2-PYR, NCM and NCM H_A_ (CSD refcode OBEQAN02); Figure S9. PXRD patterns of the solid residues recovered after 10 h in aqueous medium during kinetic solubility experiments (red), compared with patterns of fresh NCM–2-PYR and NCM H_A_ (CSD refcode OBEQAN02); Figure S10. PXRD patterns of the solid residues recovered after 48 h in aqueous medium during kinetic solubility experiments (red), compared with patterns of fresh NCM–2-PYR and NCM H_A_ (CSD refcode OBEQAN02); Figure S11. PXRD patterns of the solid residues ((a) NCM–2-PYR and (b) NCM) recovered after 72 h in aqueous medium during kinetic solubility experiments (red), compared with patterns of fresh NCM–2-PYR, NCM and NCM H_A_ (CSD refcode OBEQAN02). CCDC 2561439 contains the supplementary crystallographic data for niclosamide:2-pyrrolidone monosolvate (NCM-2-PYR) (These data can be obtained free of charge from The Cambridge Crystallographic Data Centre via www.ccdc.cam.ac.uk/structures (accessed on 11 June 2026)).

## Abbreviations

The following abbreviations are used in this manuscript:

NCM	Niclosamide
CSD	Cambridge Structural Database
APIs	active pharmaceutical ingredients
DMSO	dimethyl sulfoxide
2-PYR	2-pyrrolidone
AA	acetic acid
BENZOH	benzyl alcohol
ACT	Acetone
MeOH	Methanol
ACN	Acetonitrile
EtOH	Ethanol
EA	ethyl acetate
iPOH	Isopropanol
4-MTHP	4-methyltetrahydropyran
HXN	Hexane
PXRD	powder X-ray diffraction
DSC	differential scanning calorimetry
TGA	thermogravimetric analysis
HSM	hot-stage microscopy
SEM	scanning electron microscopy
DFT	density functional theory
PVDF	polyvinylidene fluoride
ATR	attenuated total reflectance
SA	simulated annealing
TZP	Triple-ζ plus Polarization
STO	Slater Type Orbitals
RC	regenerated cellulose
RH	relative humidity
LAG	liquid-assisted grinding
ASU	asymmetric unit
BCS	Biopharmaceutical Classification System
