# Supplementary material for: Unlocking Niclosamide Solid Forms via Mechanochemistry: Discovery of a 2-Pyrrolidone Solvate
Source: Pharmaceutics. 2026 Jul 23;18(8):906. doi: 10.3390/pharmaceutics18080906 (PMC13516761; doi:10.3390/pharmaceutics18080906)
Supplement: Supplementary file 1 [file pharmaceutics-18-00906-s001.zip › Supporting Information _Niclosamide solvate.pdf]

Supporting Information

Unlocking Niclosamide Solid Forms via Mechanochemistry: Discovery of a 2-Pyrrolidone Solvate

Ilenia D'Abbrunzo [1], Veronica Rizzetto [1], Lara Gigli [2], Nicola Demitri [2], Francesca Argentieri [1], Mauro Stener [1], Nadia Passerini [3] and Beatrice Perissutti [1,*]

[1] Department of Chemical and Pharmaceutical Sciences, University of Trieste, P.le Europa 1, 34127 Trieste, Italy; ilenia.d'abbrunzo@phd.units.it (I.D.); rizzettoveronica@gmail.com (V.R.); francesca.argentieri@phd.units.it (F.A.); stener@units.it (M.S.)
[2] Area Science Park Elettra-Sincrotrone Trieste, S.S. 14 Km 163.5 Basovizza, 34149 Trieste, Italy; lara.gigli@elettra.eu (L.G.); nicola.demitri@elettra.eu (N.D.)
[3] Department of Pharmacy and Biotechnology, University of Bologna, V. S. Donato 19/2, 40127 Bologna, Italy; nadia.passerini@unibo.it
* Correspondence: bperissutti@units.it

Contents

**Description**	**Page N°**
**Figure S1**. PXRD patterns of samples prepared via the slurry method in the presence of ACT, ACN and HXN, compared with correlated known NCM solid forms: NCM Form A (CSD refcode HEBFUR) and NCM H_A (CSD refcode OBEQAN02)	**2**
**Figure S2**. PXRD patterns of samples prepared via the slurry method in the presence of EA and EtOH, compared with correlated known NCM solid forms: NCM Form A (CSD refcode HEBFUR) and NCM Form B (CSD refcode HEBFUR01).	**3**
**Figure S3**. PXRD patterns of samples prepared via the slurry method in the presence of 4-MTHP, iPOH, AA and BENZOH, compared with correlated known NCM solid forms: NCM Form A (CSD refcode HEBFUR) and NCM H_B (CSD refcode OBEQAN).	**3**
**Figure S4**. PXRD patterns of samples prepared via the slurry method in the presence of MeOH and DMSO, compared with correlated known NCM solid forms: NCM Form A (CSD refcode HEBFUR), NCM H_A (CSD refcode OBEQAN02), NCM– MeOH (CSD refcode GOJLIC) and NCM–DMSO monosolvate (CSD refcode WOXVIS).	**4**
**Figure S5.** PXRD patterns of NCM–2-PYR at room temperature (blue), after heating to 185 °C (dark blue), and after heating to 225 °C (very dark blue), compared with the reference pattern of anhydrous NCM Form A (CSD refcode HEBFUR).	**4**
**Figure S6.** Photomicrographs of NCM–2-PYR monosolvate collected during HSM experiments (magnification 10×)	**5**
**Figure S7.** PXRD pattern of the solid recovered after the heating–cooling–heating experiment (dark blue), compared with the reference pattern of NCM Form A (CSD refcode HEBFUR).	**5**
**Figure S8.** PXRD patterns of the solid residues ((a) NCM–2-PYR and (b) NCM) recovered after 1 h in aqueous medium during kinetic solubility experiments (red), compared with patterns of fresh NCM–2-PYR, NCM and NCM H_A (CSD refcode OBEQAN02).	**6**
**Figure S9.** PXRD patterns of the solid residues recovered after 10 h in aqueous medium during kinetic solubility experiments (red), compared with patterns of fresh NCM–2-PYR and NCM HA (CSD refcode OBEQAN02).	**6**

**Figure S10.** PXRD patterns of the solid residues recovered after 48 h in aqueous medium during kinetic solubility experiments (red), compared with patterns of fresh NCM–2-PYR and NCM H_A (CSD refcode OBEQAN02).	**7**
**Figure S11.** PXRD patterns of the solid residues ((a) NCM–2-PYR and (b) NCM) recovered after 72 h in aqueous medium during kinetic solubility experiments (red), compared with patterns of fresh NCM–2-PYR, NCM and NCM H_A (CSD refcode OBEQAN02).	**7**

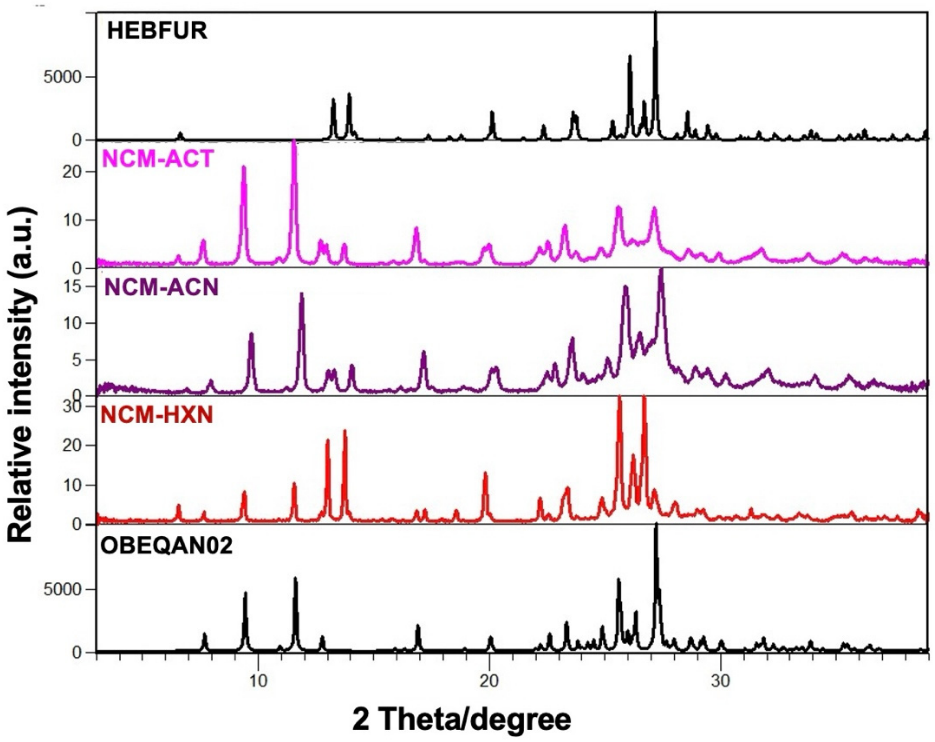

Figure S1. PXRD patterns of samples prepared via the slurry method in the presence of ACT, ACN and HXN, compared with correlated known NCM solid forms: NCM Form A (CSD refcode HEBFUR) and NCM H_A (CSD refcode OBEQAN02)

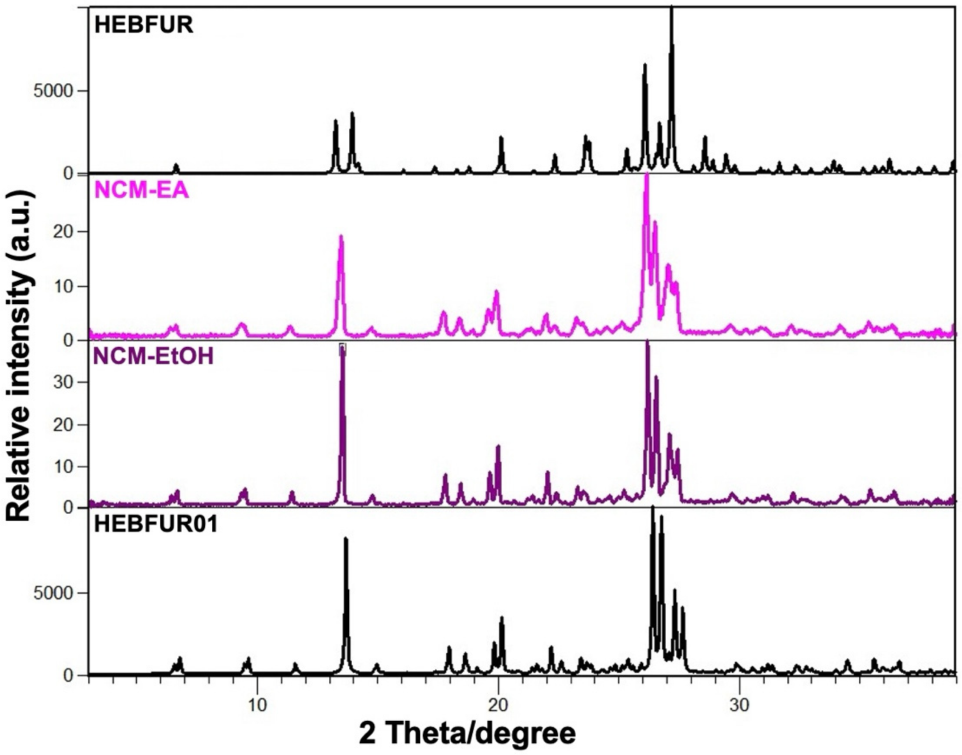

Figure S2. PXRD patterns of samples prepared via the slurry method in the presence of EA and EtOH, compared with correlated known NCM solid forms: NCM Form A (CSD refcode HEBFUR) and NCM Form B (CSD refcode HEBFUR01).

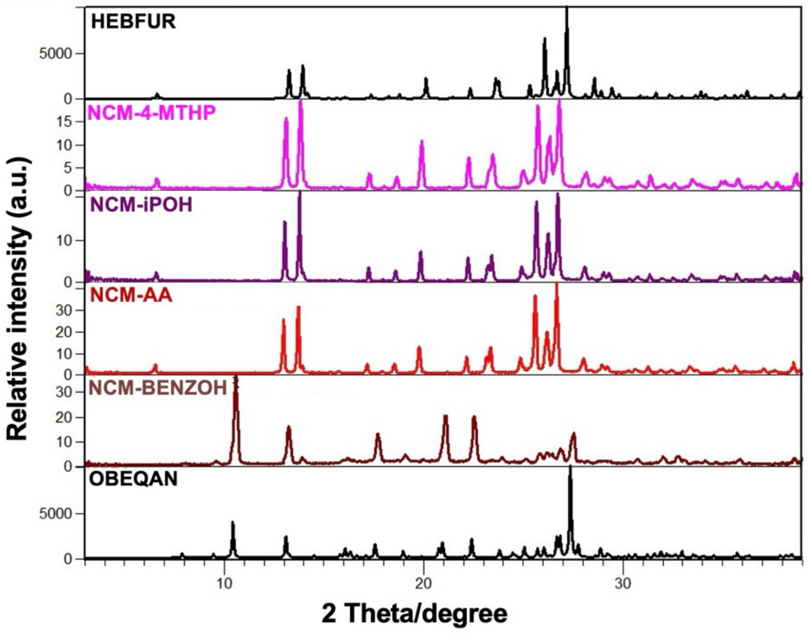

Figure S3. PXRD patterns of samples prepared via the slurry method in the presence of 4-MTHP, iPOH, AA and BENZOH, compared with correlated known NCM solid forms: NCM Form A (CSD refcode HEBFUR) and NCM H_B (CSD refcode OBEQAN).

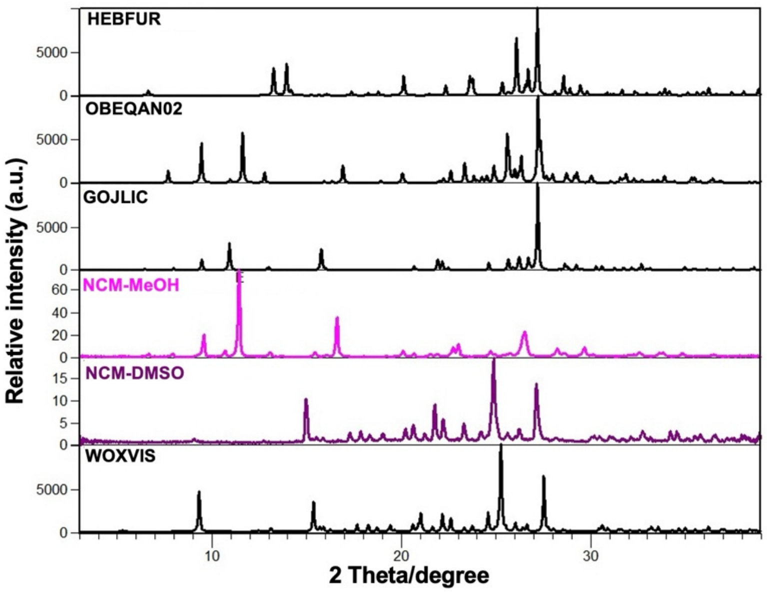

Figure S4. PXRD patterns of samples prepared via the slurry method in the presence of MeOH and DMSO, compared with correlated known NCM solid forms: NCM Form A (CSD refcode HEBFUR), NCM H_A (CSD refcode OBEQAN02), NCM– MeOH (CSD refcode GOJLIC) and NCM–DMSO monosolvate (CSD refcode WOXVIS).

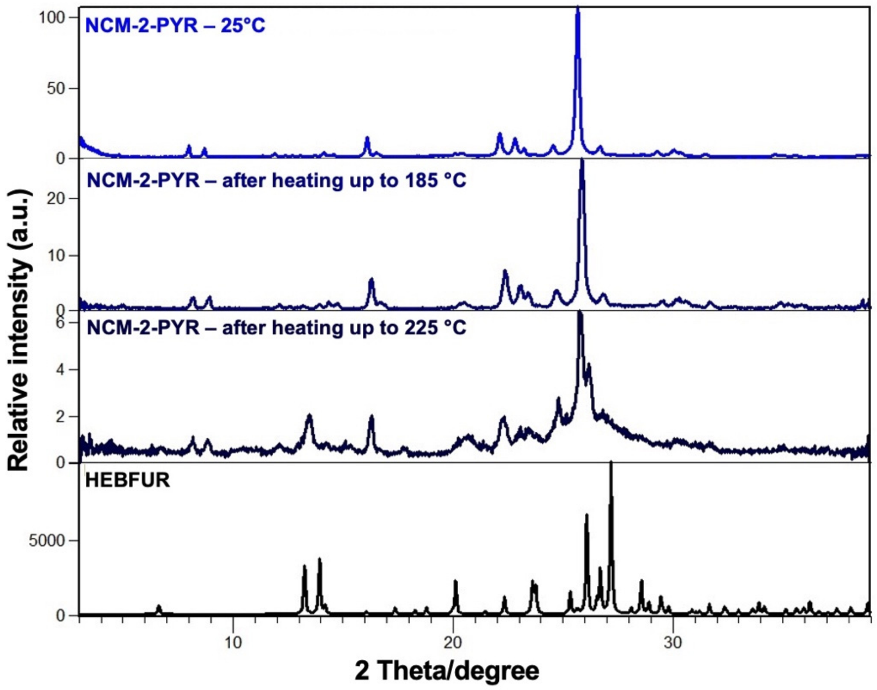

Figure S5. PXRD patterns of NCM–2-PYR at room temperature (blue), after heating to 185 °C (dark blue), and after heating to 225 °C (very dark blue), compared with the reference pattern of anhydrous NCM Form A (CSD refcode HEBFUR).

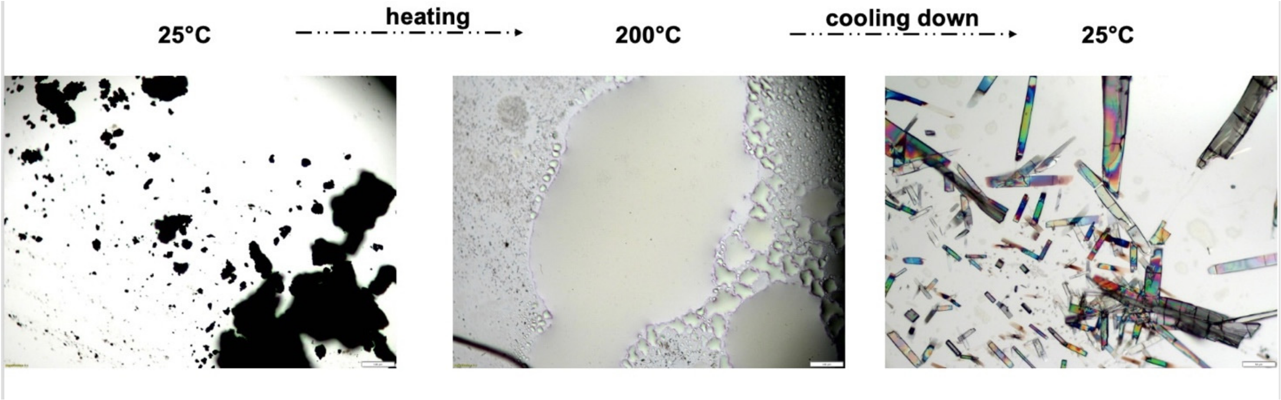

Figure S6. Photomicrographs of NCM–2-PYR monosolvate collected during HSM experiments (magnification 10×).

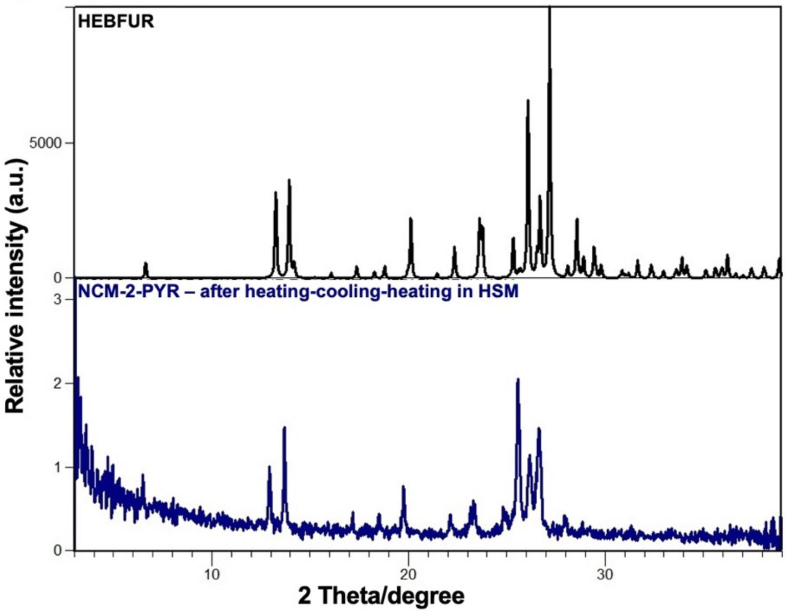

Figure S7. PXRD pattern of the solid recovered after the heating–cooling–heating experiment (dark blue), compared with the reference pattern of NCM Form A (CSD refcode HEBFUR).

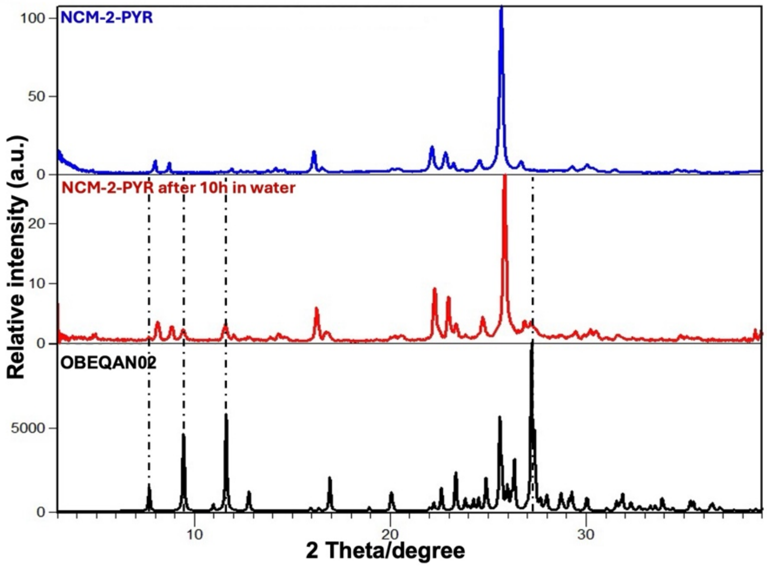

Figure S8. PXRD patterns of the solid residues recovered after 10 h in aqueous medium during kinetic solubility experiments (red), compared with patterns of fresh NCM–2-PYR and NCM H_A (CSD refcode OBEQAN02).

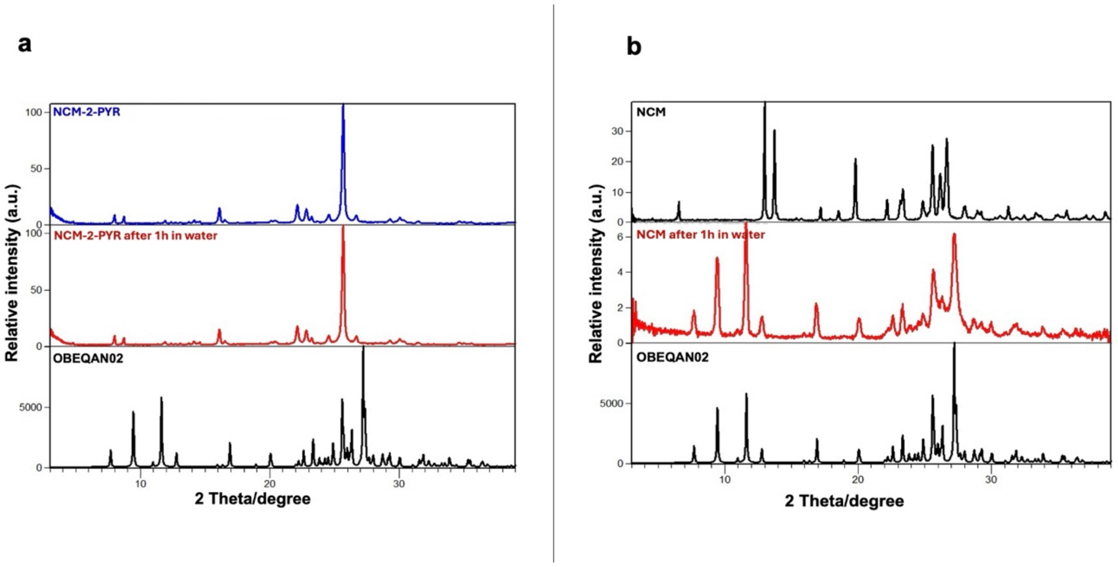

Figure S9. PXRD patterns of the solid residues ((a) NCM–2-PYR and (b) NCM) recovered after 1 h in aqueous medium during kinetic solubility experiments (red), compared with patterns of fresh NCM–2-PYR, NCM and NCM H_A (CSD refcode OBEQAN02).

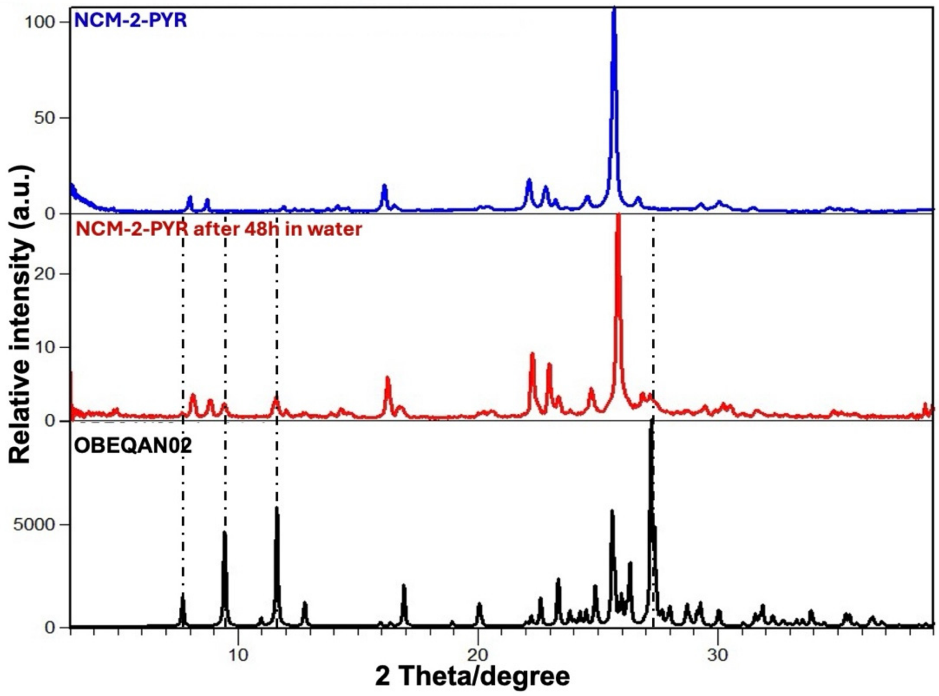

Figure S10. PXRD patterns of the solid residues recovered after 48 h in aqueous medium during kinetic solubility experiments (red), compared with patterns of fresh NCM–2-PYR and NCM H_A (CSD refcode OBEQAN02).

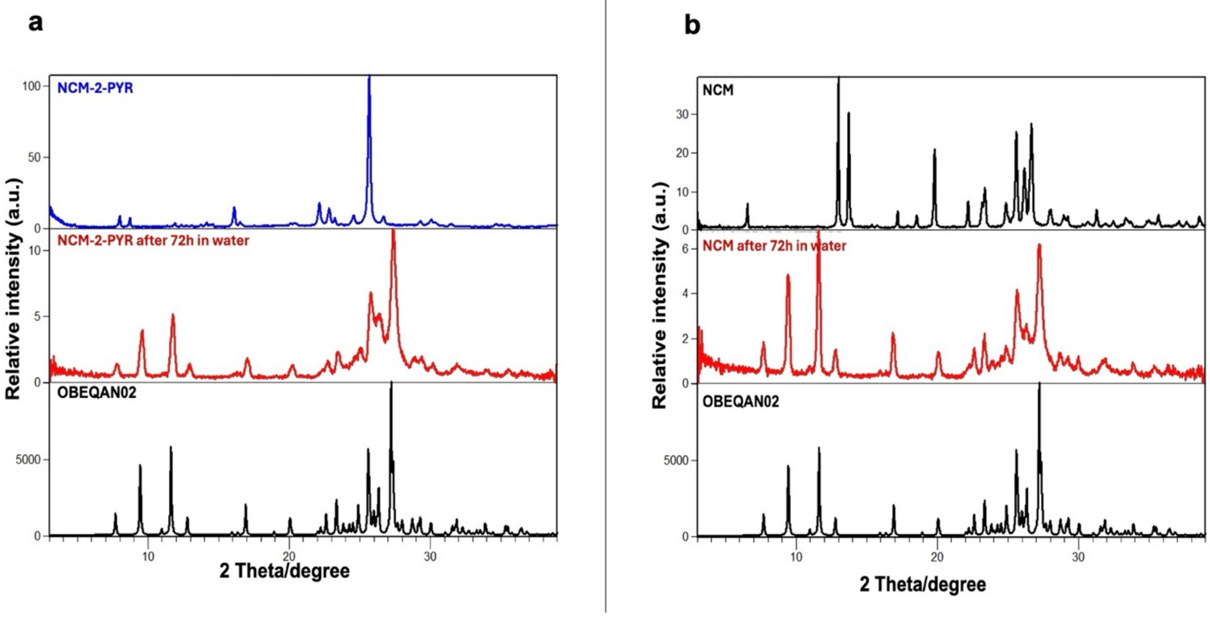

Figure S11. PXRD patterns of the solid residues ((a) NCM–2-PYR and (b) NCM) recovered after 72 h in aqueous medium during kinetic solubility experiments (red), compared with patterns of fresh NCM–2-PYR, NCM and NCM H_A (CSD refcode OBEQAN02).
